# Protective Property of Scutellarin Against Liver Injury Induced by Carbon Tetrachloride in Mice

**DOI:** 10.3389/fphar.2021.710692

**Published:** 2021-08-05

**Authors:** Zhimin Miao, Yong Lai, Yingying Zhao, Lingmin Chen, Jianeng Zhou, Chunyan Li, Yan Wang

**Affiliations:** College of Pharmacy, Dali University, Dali, China

**Keywords:** scutellarin, liver injury, transcriptomics, nontargeted metabolomics, 16S rRNA amplicon sequencing

## Abstract

Liver injury is a clinical disorder caused by toxins, drugs, and alcohol stimulation without effective therapeutic approaches thus far. Scutellarin (SCU), isolated from the edible herb *Erigeron breviscapus* (Vant.) Hand. -Mazz. showed potential hepatoprotective effects, but the mechanisms remain unknown. In this study, transcriptomics combined with nontargeted metabolomics and 16S rRNA amplicon sequencing were performed to elucidate the functional mechanisms of SCU in carbon tetrachloride (CCl_4_)–induced liver injury in mice. The results showed that SCU exerted potential hepatoprotective effects against CCl_4_-induced liver injury by repressing CYP2E1 and IκBα/NF-κB signaling pathways, modulating the gut microbiota (especially enriching *Lactobacillus*), and regulating the endogenous metabolites involved in lipid metabolism and bile acid homeostasis. SCU originates from a functional food that appears to be a promising agent to guard against liver injury.

## Introduction

Liver injury is caused by a constellation of risk factors such as drug abuse and excessive alcohol consumption and can initiate cascades of pathophysiological processes, which subsequently contribute to the development of hepatosteatosis, hepatitis, and hepatic fibrosis, among others (Kremer et al., 2006; Chen et al., 2018; Qu et al., 2020). Liver diseases that develop from liver injury impose major burdens of costs and have attracted considerable attention worldwide. However, treatment strategies for liver injury remain extremely limited.

Dietary flavonoids, including anthocyan, hyperoside, silymarin, and luteolin, are known for their beneficial effects on health and active roles in the prevention and treatment of a variety of diseases, such as cardiovascular disease, liver injury, fibrosis, and cancer (Gonçalves et al., 2009; Imran et al., 2019; Rahman et al., 2021; Zhang et al., 2021). *Erigeron* *breviscapus* (Vant.) Hand. -Mazz. is a Chinese ethnomedicine mainly distributed in Yunnan. The whole herb of *E*. *breviscapus* is edible and has been used as functional herb tea in Yunnan. The Bai minority often stew eggs with *E*. *breviscapus* (Liu et al., 2008). In addition, the whole herb of *E*. *breviscapus* has been applied in the treatment of cerebral embolism, arachnoiditis, hemiplegia, and coronary artery disease for centuries in folk medicine (Liu et al., 2017; Zhu et al., 2018). The main active extract of *E*. *breviscapus* is scutellarin (SCU), a flavonoid compound with hepatoprotective potential. However, the mechanisms by which SCU ameliorates liver injury have remained unknown until relatively recently.

Of note, the bioavailability of SCU is exceptionally low. In healthy volunteers and rats, the oral bioavailability of SCU was found to be merely 2.2 and 0.67%, respectively (Wang and Ma, 2018). The effects of SCU are in notable contrast to its poor bioavailability. The gut microbiota that harbors within the host gut comprises over 100 trillion bacteria. With the advent of 16 S rRNA sequencing-based taxonomic profiling and the development of germ-free models, investigators have been appreciating the substantial effects of the gut microbiota on clinical disorders. Duan et al. (2019) reported that cytolytic *Enterococcus faecalis* was linked with the mortality of patients with alcoholic hepatitis and that bacteriophages targeting cytolytic *E. faecalis* abolished ethanol-induced liver disease in humanized mice. Furthermore, supplementation with probiotics such as *Lactobacillus* and *Bifidobacterium* effectively improved hepatic disorders in mouse models (Gu et al., 2020; Zhang et al., 2020). Drugging gut microbes may be a promising method to mitigate liver diseases. Currently, numerous phytonutrients with low bioavailability have been proven to exert their pharmacological effects by remodeling gut microbiota, such as raising the relative abundance of probiotics (Dey 2019; Zeng et al., 2020). Thus, modulation of gut microbiota may be one of the mechanisms of the hepatic effects of SCU.

In this study, we utilized transcriptomic analysis using RNA sequencing (RNA-seq) to identify the signaling pathways in the liver that are modified by SCU, and the results showed that expression of the *nf-κb* and *cyp2e1* genes were significantly dampened. We generated a carbon tetrachloride (CCl_4_)–induced liver injury mouse model to determine whether the hepatic effects of SCU were mediated by inhibiting CYP2E1 and NF-κB pathways. We performed taxonomic profiling based on 16 S rRNA sequencing to identify the specific genus associated with the hepatoprotective effects of SCU and observed that *Lactobacillus* was significantly enriched upon SCU treatment. Finally, we performed nontargeted metabolomics analysis using UHPLC-Q-Exactive MS/MS to explore the potential mechanisms of SCU in improving CCl_4_-induced liver injury and observed that endogenous metabolites involved in linoleic acid metabolism, biosynthesis of unsaturated fatty acids, bile secretion, and retinol metabolism were significantly altered in feces and liver tissues upon SCU treatment. These data indicate that SCU derived from a functional food appears to be a promising agent to protect against liver injury.

## Materials and Methods

### Chemicals

Scutellarin (purity >98%, cat# HB20121201) was purchased from Yunnan Plant Pharmaceutical Co., Ltd (Kunming, Yunnan, China). Bifendate (cat# H33021305) was purchased from Yunnan Jianzhijia Co., Ltd (Yunnan, China). CCl_4_ (cat# 80123318) and olive oil (cat# 69018028) were purchased from Sinoreagent (Shanghai, China).

### Animal and Experimental Design

SPF-grade BALB/c mice (male, 18–22 g, 8 weeks old) were obtained from Tianqin Biotechnology Co., Ltd (Hunan, China). Before the experiment, mice were acclimatized to the environment (20 ± 3 °C, 12 h light/dark cycle) with free access to food and water for 1 week. The experiments were carried out in accordance with the Animal Welfare Guidelines and approved by the Animal Care and Use Committee of Dali University (No. 2017–1201).

**Experiment 1**: BALB/c mice were orally administrated either 0.5% CMC-Na or SCU (0.12 mmol/kg; suspended in 0.5% CMC-Na) for 5 weeks (*n* = 10 per group). Mice were harvested following the last gavage. Liver tissues were collected for RNA-seq.

**Experiment 2**: BALB/c mice were intraperitoneally injected with either CCl_4_ (1 ml/kg; 1:9 dilution with olive oil) or an equal volume of olive oil three times per week, plus daily gavage of 0.5% CMC-Na or SCU (0.03, 0.06, and 0.12 mmol/kg) or bifendate (0.4 mmol/kg) for 5 weeks (*n* = 10 per group). Mice were euthanized following the last injection of CCl_4_. Blood samples, liver tissues, and feces of each mouse were collected.

### Transcriptome Analysis Based on RNA-Seq

Transcriptome analysis between the control and SCU groups was conducted by Shenggong Bioengineering Co., Ltd (Shanghai, China). Briefly, RNA was isolated from snap-frozen liver tissues using TRIzol (Ambion, United States ) and assessed for quantity using an Agilent 2100 Bioanalyzer (Agilent Technologies, United States). After purification and fragmentation, RNA was reverse transcribed into cDNA using a SMART PCR cDNA Synthesis Kit (Clontech, Takara Bio). Clustered 300–400 bp libraries were validated using an Agilent 2,100 Bioanalyzer (Agilent Technologies, United States ), quantified using a Qubit fluorometer (Thermo Fisher Scientific, United States ), and then sequenced on the Illumina HiSeq 3,000 platform. Transcript abundance was estimated using StringTie and known gene models. Differential expression analyses were performed using the DESeq2. KEGG pathway analysis was performed by clusterProfiler. The raw data were deposited into the NCBI Sequence Read Archive (SRA) database (accession number PRJNA736950, https://www.ncbi.nlm.nih.gov/bioproject/PRJNA736950).

### Biochemical Assay

Blood samples and a portion of the liver tissues were collected for biochemical assays. Blood samples were centrifuged (3,000 rpm, 4 °C, 10 min) to obtain serum, and alanine aminotransferase (ALT), aspartate aminotransferase (AST), albumin (ALB), and total bilirubin (TBIL) in the serum were determined by the corresponding kits. Liver tissues were homogenized with PBS and then centrifuged (10,000 rpm, 4 °C, 25 min) to obtain supernatant. The activity of superoxide dismutase (SOD) and the content of malondialdehyde (MDA) were determined by commercial kits. All kits for biochemical assays were purchased from Nanjing Jiancheng Bioengineering Institute (Nanjing, China).

### Histopathology Assay

A portion of the liver tissues was preserved for histopathology assay. Briefly, 4 μm thick liver paraffin-embedded sections were paraformaldehyde-fixed and stained with hematoxylin-eosin (H and E). Images were obtained at 200X, and hepatic lesions were based on assessment of hepatocyte necrosis and hepatic inflammatory cell infiltration.

### TUNEL Assay

Hepatocyte apoptosis was determined by the DeadEnd^TM^ Fluorometric TUNEL System (Promega, Wisconsin, United States). Briefly, 4 μm thick liver paraffin-embedded sections were digested by 20 μg/ml proteinase K and then incubated with TdT reaction mix. Finally, the sections were stained with propidium iodide. Images were obtained at 200X under a fluorescence microscope (Olympus, Tokyo, Japan), and quantification of fluorescence intensity was performed using ImageJ.

### Immunohistochemical Assay

Liver paraffin-embedded sections (4 μm thick) were first incubated with primary antibody and then incubated with HRP-conjugated secondary antibody. Finally, the sections were stained with a DAB substrate. The positive expression was measured by ImageJ.

### RT-qPCR

A portion of the liver tissues was snap frozen in liquid nitrogen for RT-qPCR. Liver tissues were disrupted in TRIzol (Invitrogen, United States), and RNAs were prepared according to the TRIzol manufacturer’s protocol. RT-qPCR was performed using TB Green^®^ Premix Ex Taq^TM^ II (Takara Bio, Inc., Shiga, Japan), and the primers were as follows:

IL-6 sense: 5′-CTG​CAA​GAG​ACT​TCC​ATC​CAG-3′, and antisense: 5′-AGT​GGT​ATA​GAC​AGG​TCT​GTT​GG-3′; IL-1β sense: 5′-TGT​GAA​ATG​CCA​CCT​TTT​GA-3′, and antisense: 5′-GGT​CAA​AGG​TTT​GGA​AGC​AG-3′; TNF-α sense: 5′-CAG​GCG​GTG​CCT​ATG​TCT​C-3′, and antisense: 5′-CGA​TCA​CCC​CGA​AGT​TCA​GTA​G-3′; CYP2E1 sense: 5′-TTT​CCC​TAA​GTA​TCC​TCC​GTG​AC-3′, and antisense: 5′-CTT​AAT​CGA​AGC​GTT​TGT​TGA-3′; and GAPDH sense: 5′-GGT​TGT​CTC​CTG​CGA​CTT​CA-3′, and antisense: 5′-TGG​TCC​AGG​GTT​TCT​TAC​TCC-3′. GAPDH was served as an internal control. Fold change was calculated using the 2^−ΔΔCt^ method.

### Western Blot

A portion of the liver tissues was snap frozen in liquid nitrogen for the Western blot assay. Total proteins of liver tissues were extracted with RIPA lysis buffer and quantified by a BCA kit (Solarbio, Beijing, China). Forty micrograms of protein was electrophoresed on 10% SDS gels and transferred to polyvinylidene fluoride membranes. The membranes were incubated with primary antibodies overnight at 4 °C and then incubated with HRP-conjugated secondary antibodies for 1 h at room temperature. The blots were imaged using a G:BOX gel imaging system (Syngene, Cambridge, United Kingdom). The densitometric analysis was performed using ImageJ. Data were normalized to GAPDH.

### Bioinformatics Assay

Total bacterial DNA from fecal samples was isolated using a QIAamp DNA Stool Kit (Qiagen, Valencia, United States). The yield and quality of DNAs were measured by a Nanodrop ND 1,000 Spectrophotometer (Thermo Fisher Scientific, United States) and 0.8% agarose gel electrophoresis, respectively. The V3-V4 region of the bacterial 16 S rRNA gene was amplified by PCR (forward primer: 5′-ACT​CCT​ACG​GGA​GGC​AGC​A-3′ and reverse primer: 5′-GGACTACHVGGGTWTCTAAT-3′). PCR products were purified with Vazyme VAHTS™ DNA Clean Beads (Vazyme, Shanghai, China) and quantified using a PicoGreen dsDNA Assay Kit (Invitrogen, United States ). The sequencing service was provided by Personal Biotechnology Co., Ltd (Shanghai, China). The alpha diversity, including the Chao1 and Shannon indices, was calculated using OTUs in QIIME (Denver, United States ). Beta diversity was visualized by principal coordinate analysis (PCoA). The genus difference was measured using the Z score. The correlation between genus and liver injury indicators was analyzed using Spearman’s correlation analysis. The prediction of microbiome function was analyzed by PICRUSt, based on the KEGG database. The raw data were deposited into the NCBI Sequence Read Archive (SRA) database (accession number PRJNA736871, https://www.ncbi.nlm.nih.gov/bioproject/?term = PRJNA736871).

### Nontargeted Metabolomics Based on UHPLC-Q-Exactive MS/MS

Nontargeted metabolomics was conducted by Personal Biotechnology Co., Ltd (Shanghai, China). Chromatography was performed on an Ultimate 3000 UPLC system (Thermo Fisher Scientific, United States ). An ACQUITY UPLC BEH C_18_ (100 × 2.1 mm, 1.7 μm, Waters, United States ) was adopted for separation with the column temperature maintained at 40 °C and the flow rate was 0.3 ml/min. The mobile phase consisted of (A) 0.1% formic acid and (B) acetonitrile in a gradient elusion as: 0–0.5 min, 5% B; 0.5–1.0 min, 5% B; 1.0–9.0 min, 5–100% B; 9.0–12.0 min, 100% B; and 12.0–15.0 min, 5% B. The injection volume of each sample was 5 μl. Mass detection was carried out on a Q-Exactive high resolution mass spectrometer (Thermo Fisher Scientific, United States) coupled with an electrospray ionization (ESI) source. The ESI source conditions were as follows: ion source gas 1 (Gas1), 60; ion source gas 2 (Gas2), 60; curtain gas (CUR), 30; source temperature, 320 °C; ion spray voltage floating (ISVF), ± 3500 V (positive and negative modes); MS scan m/z range, 80–1200 Da; product ion scan resolution, 17,500; MS scan accumulation time, 0.20 s/spectra; and product ion scan accumulation time, 0.05 s/spectra. Secondary mass spectrometry was used for information-dependent acquisition (IDA) in a high-sensitivity model, and the conditions were as follows: declustering potential (DP), ±60 V (positive and negative modes); collision energy, 35 ± 15 eV; excluding isotopes within 4 Da; and candidate ions to monitor per cycle, 6.

The raw data were analyzed with Compound Discoverer 3.0 (Thermo Fisher Scientific, United States), including peak extraction, alignment, correction, and standardization. The structure of metabolites was identified by accurate mass matching (<25 ppm) and secondary spectrum matching. SIMCA-P 14.1 software (Umetrics, Umea, Sweden) was used for pattern recognition. The data were preprocessed by Pareto scaling and analyzed by multidimensional statistical methods containing unsupervised principal component analysis (PCA), supervised partial least squares discriminant analysis (PLS-DA), and orthogonal partial least squares discriminant analysis (OPLS-DA). One-dimensional statistical analysis included Student’s t-test and multiple of variation analysis. In this study, the altered metabolites with variable importance for projection (VIP) > 1.00 and *p* < 0.05 among the control, model, and SCU groups were selected as potential biomarkers for liver injury.

### Statistical Analysis

All data were presented as the mean ± SD. Statistical significance was determined by one-way analysis of variance (ANOVA) followed by Dunnett’s multiple comparison test. All data were considered statistically significant at *p* < 0.05.

## Results

### SCU Displayed Hepatoprotective Potential

**FIGURE1:**
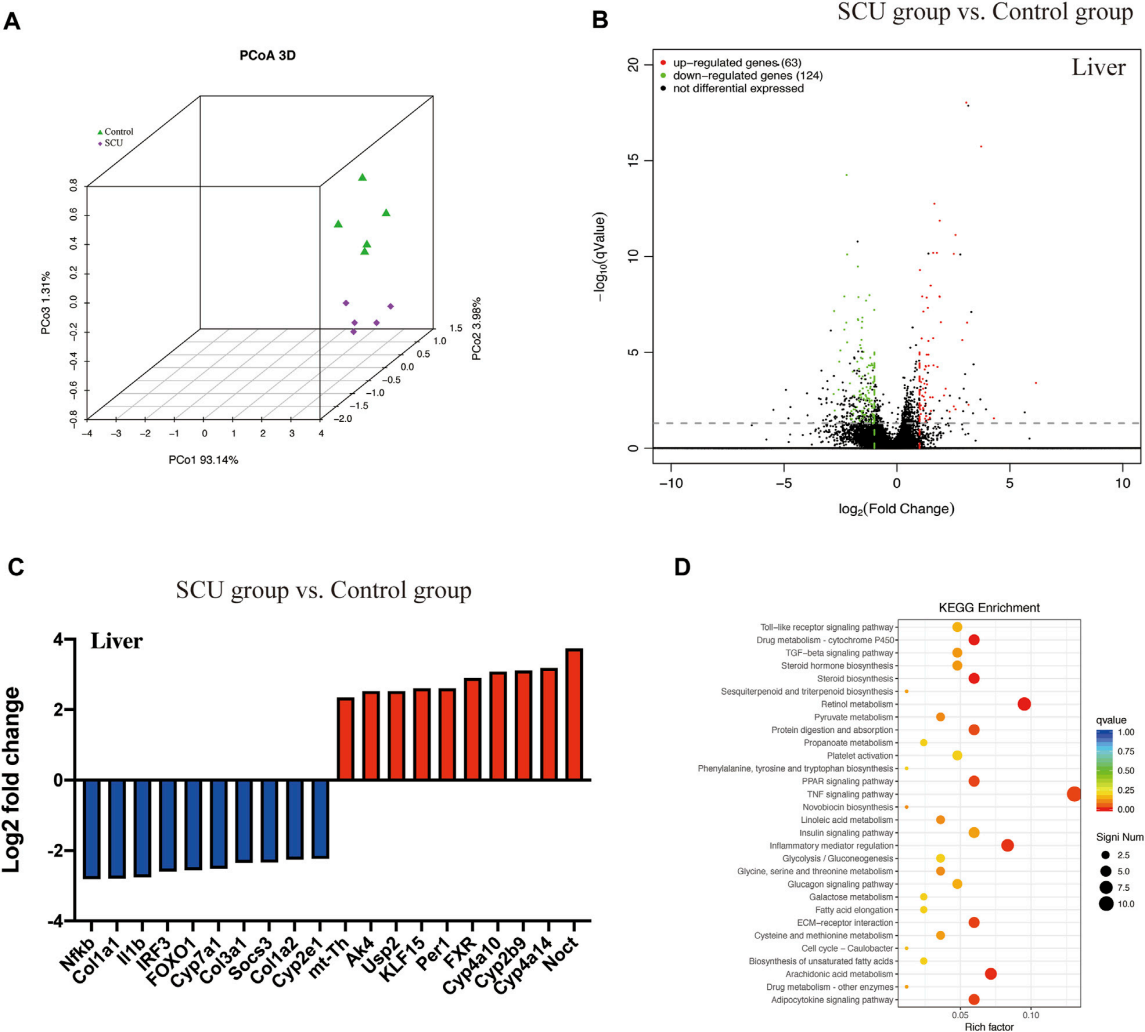
SCU displayed hepatoprotective potential. **(A)** Principal coordinate analysis (PCoA). **(B)** Volcano plot of transcripts. **(C)** Top 20 transcripts in liver tissues. **(D)** KEGG pathway. (*n* = 5).

Transcriptomic analysis demonstrated a significantly altered profile in the hepatic transcriptome of the SCU group relative to the control group (Figures 1A,B). The *nf-κb* gene, a master regulator of the cellular inflammatory response, was significantly dampened. Furthermore, we also observed that the *cyp2e1* gene, a toxicological protein that regulates alcohol, acetaminophen, and CCl_4_ metabolism, was inhibited in the SCU-treated group (Figure 1C). The KEGG pathway analysis showed that 30 signaling pathways were significantly changed upon SCU treatment and revealed a regulatory network involved in the anti-inflammatory response and drug metabolism (Figure 1D). Collectively, these data indicate that SCU has significant influences on the hepatic inflammatory response and displays hepatoprotective potential.

### SCU Protected Against CCl4-Induced Liver Injury in Mice

**FIGURE 2:**
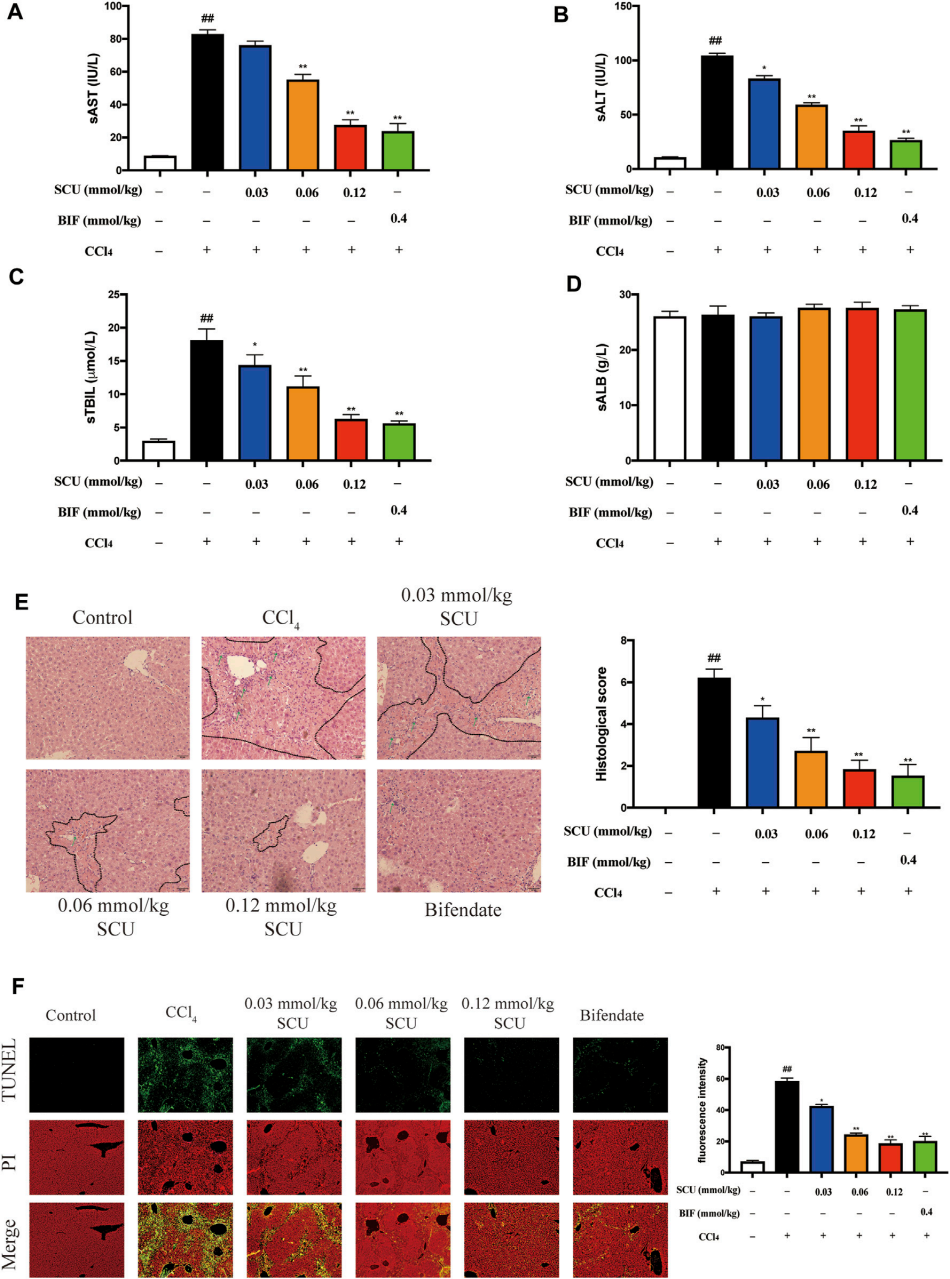
SCU protected against CCl4-induced liver injury in mice. Serum (**A)** AST, **(B)** ALT, **(C)** TBIL, and **(D)** ALB levels. **(E)** H and E staining (dotted line parts represent the necrosis area; arrows represent the inflammatory cell infiltration). **(F)** TUNEL staining. Scale bar 50 μm, magnification 200×. (*n* = 5). ^##^ *p* < 0.01 vs. control group; ^*^ *p* < 0.05, ^**^ *p* < 0.01 vs. CCl_4_ group.

We sought to evaluate the hepatoprotective effects of SCU in a mammalian model of CCl_4_ toxicity. As shown in Figures 2A–C, serum AST, ALT, and TBIL levels were significantly elevated in the CCl_4_ group but were markedly blunted in the SCU-treated groups. Serum ALB levels were not significantly different among the six groups (Figure 2D). In agreement with these results, CCl_4_-treated mice had a large amount of centrilobular necrosis, inflammatory cell infiltration, and hepatocyte apoptosis, whereas liver injury in the SCU-treated groups was significantly attenuated (Figures 2E,F).

### SCU Inhibited CYP2E1 and NF-κB in Mice with CCl4 Hepatotoxicity

**FIGURE 3:**
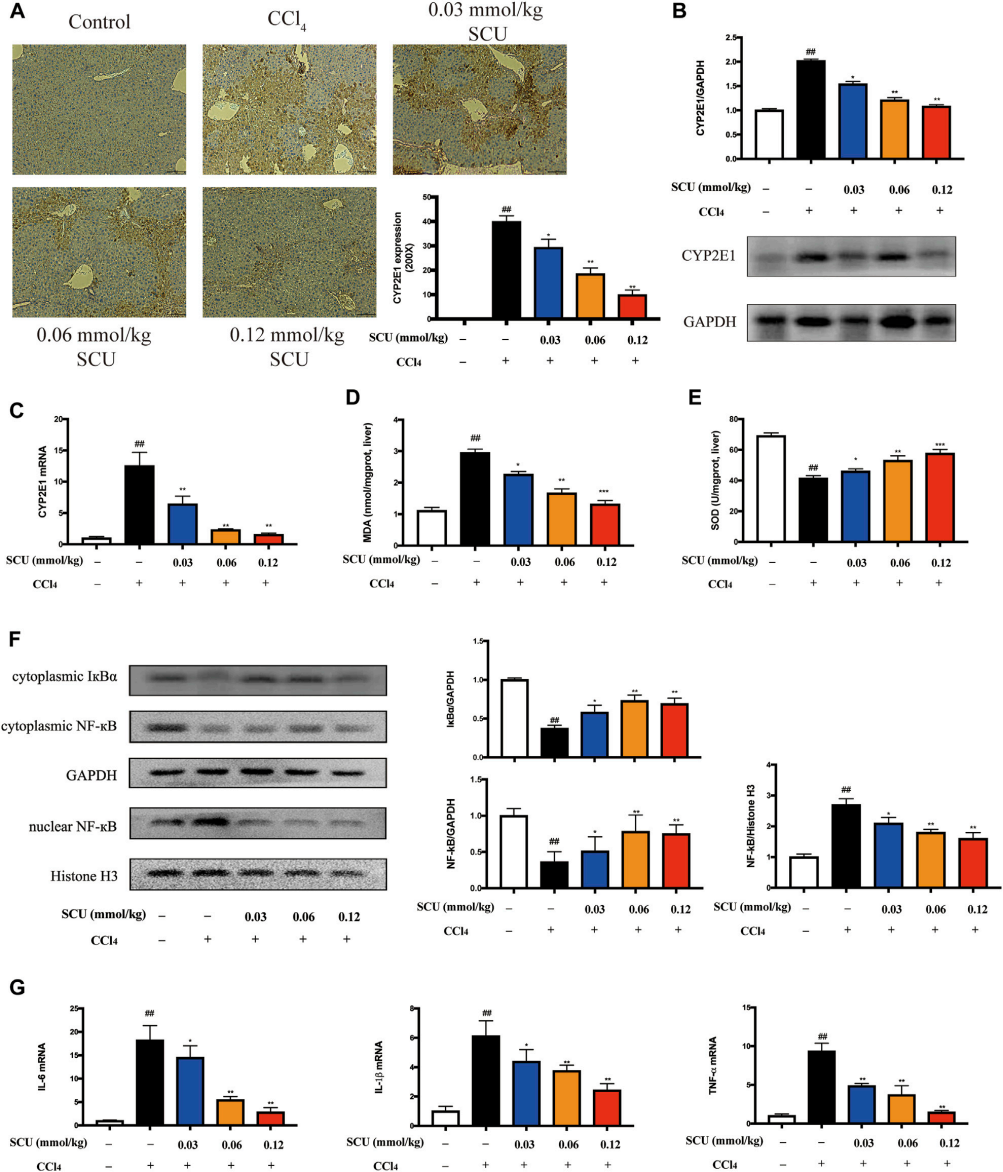
SCU inhibited CYP2E1 and NF-κB in mice with CCl_4_ hepatotoxicity. **(A)** Immunohistochemistry (yellow area, scale bar 50 μm, magnification 200×), **(B)** Western blot, and **(C)** RT-qPCR of CYP2E1. Hepatic content of **(D)** MDA and **(E)** SOD. **(F)** Western blot of IκBα and NF-κB. **(G)** Hepatic transcripts of IL-6, IL-1β, and TNF-α (*n* = 5). ^##^ *p* < 0.01 vs. control group; ^*^ *p* < 0.05, ^**^ *p* < 0.01 vs. CCl_4_ group.

We next sought to corroborate that the hepatic effects of SCU are associated with CYP2E1 and NF-κB in a mouse model of CCl_4_ hepatotoxicity. CCl_4_ is mainly metabolized by hepatic CYP2E1 to generate free radicals, which can trigger oxidative stress and indirectly induce inflammatory responses (Zhang et al., 2020). CCl_4_ challenge significantly increased hepatic CYP2E1 expression (Figures 3A–C) and led to an increase in hepatic MDA (Figure 3D) and a decrease in hepatic SOD (Figure 3E). Oxidative stress mediated by CYP2E1 was significantly improved by SCU treatment (Figures 3A–E). CCl_4_ can also directly stimulate inflammatory responses. NF-κB is a key transcription factor that regulates the expression of inflammatory genes, playing a critical role in the inflammatory response (Ma et al., 2015). Based on the results in Figure 1C, NF-κB is also a potential target of SCU. To verify the above result, we detected the expression of IκBα and NF-κB in the liver. CCl_4_ poisoning dramatically downregulated the cytoplasmic expression of IκBα and NF-κB and upregulated nuclear NF-κB. The IκBα/NF-κB signaling pathway was significantly inhibited by SCU in a dose-dependent manner (Figure 3F). IL-6, IL-1β, and TNF-α are key inflammatory cytokines regulated by the IκBα/NF-κB signaling pathway. To confirm that the inhibition of the IκBα/NF-κB signaling pathway was a result of decreased production of these three inflammatory cytokines, we detected IL-6, IL-1β, and TNF-α transcripts in the liver. Compared with the control group, hepatic mRNA levels of IL-6, IL-1β, and TNF-α were remarkably increased in the CCl_4_ model group. These increases were significantly reduced by SCU treatment (Figure 3G).

### SCU Modulated Gut Microbiota

**FIGURE 4:**
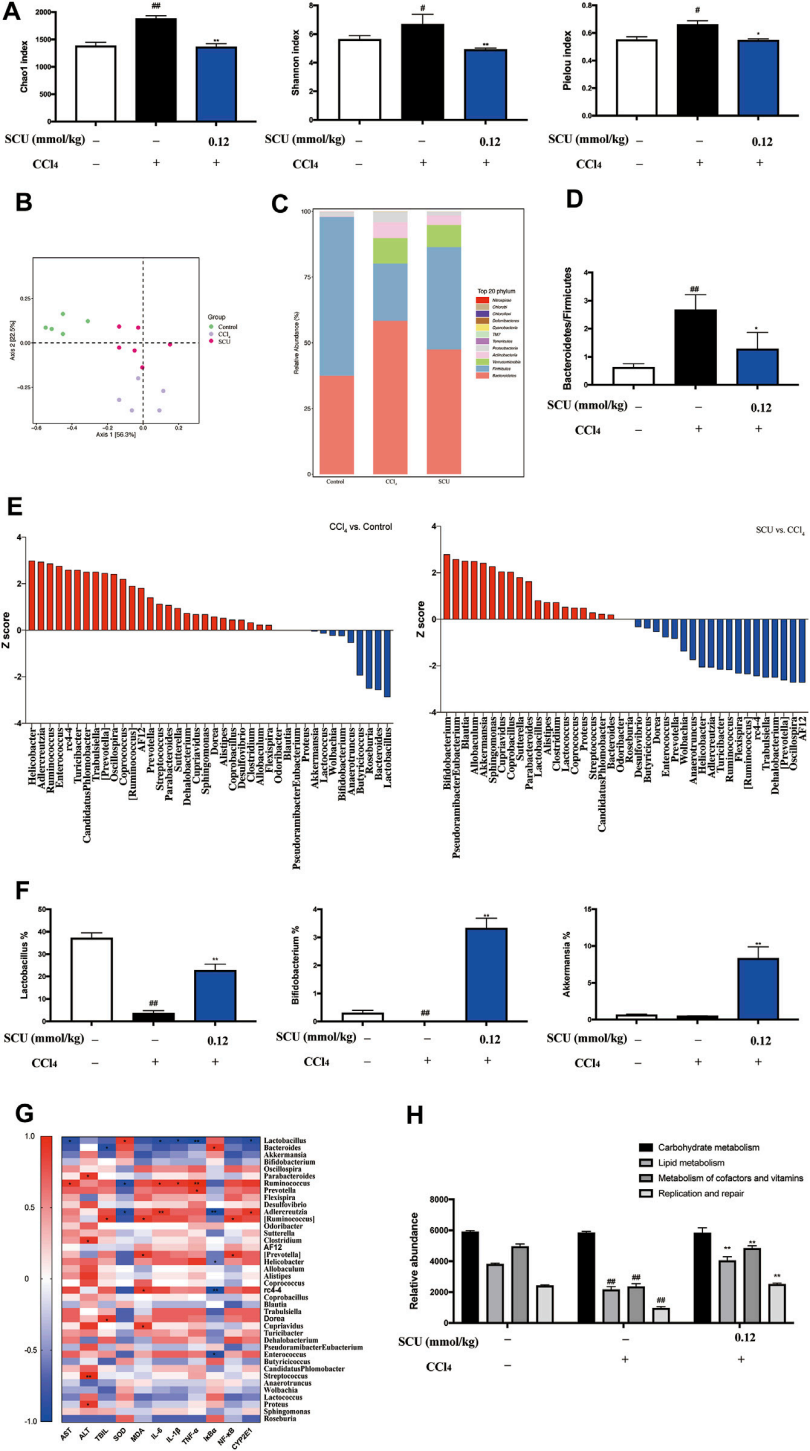
SCU modulated gut microbiota **(A)** Chao1, Shannon, and Pielou index. **(B)** PCoA of gut microbiota. **(C)** Bacterial taxonomic at phylum level. **(D)** Bacteroidetes-to-Firmicutes ratio. **(E)** Z score analysis. **(F)** Selected three genera. **(G)** Spearman correlation analysis between gut microbiota and liver injury indicators, (*, **) indicates a significant correlation (*p* < 0.05, *p* < 0.01). **(H)** Prediction of microbiome function based on the KEGG database. (*n* = 5). ^#^ *p* < 0.05, ^##^ *p* < 0.01 vs. control group; ^*^ *p* < 0.05, ^**^ *p* < 0.01 vs. CCl_4_ group.

The gut microbiota has been recognized as a critical assistant in the pharmacological effects of phytonutrients with low bioavailability. As the above data indicated that the high-dose SCU group (0.12 mmol/kg) exhibited better hepatoprotective effects in the CCl_4_ liver injury model, gut microbiota in fecal samples from the control, CCl_4_, and high-dose SCU groups were analyzed in this part. We employed Chao1, Shannon, and Pielou indices to assess the richness, diversity, and evenness of gut microbiota. CCl_4_ stimulation significantly increased the three indices, which was restored by SCU treatment (Figure 4A). We observed a distinct clustering of microbiota composition for the control, the CCl_4_ model, and the SCU groups using PCoA (Figure 4B). In addition, we analyzed the degree of bacterial taxonomic similarity at the phylum level to assess the overall gut microbiota composition shift in the control, the CCl_4_ model, and the SCU groups (Figure 4C). The *Bacteroidetes-to-Firmicutes* ratio was significantly increased in the CCl_4_ model group (Figure 4D) and was decreased in the SCU group (Figure 4D). The Z score was further used to identify the specific genera that were altered by CCl_4_ and SCU treatment (Figure 4E). The collective genera among the three groups were selected in accordance with the Z score >2 and relative abundance >3%. The relative abundances of *Lactobacillus*, *Bifidobacterium*, and *Akkermansia* were significantly blunted in the CCl_4_ model group relative to the control group. SCU treatment significantly elevated the relative abundance of these genera (Figure 4F). Moreover, Spearman’s correlation analysis revealed that only *Lactobacillus* was positively or negatively correlated with liver injury features, including SOD, AST, IL-6, IL-1β, TNF-α, NF-κB, and CYP2E1, among the three genera (Figure 4G). Through PICRUSt analysis based on the KEGG database, it was found that metabolic pathways, including lipid metabolism, metabolism of cofactors and vitamins, and replication and repair, were restored upon SCU treatment (Figure 4H).

### Metabolite Profiling Analysis After SCU Treatment

Untargeted feces and liver tissue metabolomics analysis were employed to further evaluate the ameliorative effects of SCU on CCl_4_-induced liver injury. The total ion current (TIC) of feces and liver tissue samples from the control, the CCl_4_ model, and the SCU groups in both positive and negative modes are shown in Supplementary Figure 1. PCA and supervised OPLS-DA revealed a clear separation among the control, the CCl_4_ model, and the SCU-treated groups (Supplementary Figure 2). In addition, all OPLS-DA models presented excellent stability among the control, the CCl_4_ model, and the SCU-treated feces and liver tissue samples (Supplementary Figure 2). No overfitting was observed based on the results of permutation tests (Supplementary Figure 3).

Variables from the OPLS-DA model with a VIP >1 and *p* < 0.05 were classified as differentially regulated metabolites that could discriminate among the control, the CCl_4_ model, and the SCU groups. In fecal samples, we identified seven altered metabolites involved in linoleic acid metabolism, biosynthesis of unsaturated fatty acids, and bile secretion (Tables 1, 2). In liver tissue samples, we identified 11 altered metabolites involved in retinol metabolism (Tables 3, 4).

**TABLE 1:** Altered metabolites in feces among the control, CCl_4_, and the SCU group.

Metabolites	CCl_4_ vs. control group						SCU vs. CCl_4_ group			
	MW	*p* value	VIP score	Change fold	Trend		*p* value	VIP score	Change fold	Trend
Quinaprilat	410.18	0.016	1.03	2.60	↓^a^		0.004	1.13	3.19	↑^b^
3-Phenylpropanoic acid	150.06	0.025	1.13	3.04	↓^a^		0.03	1.00	2.24	↑^a^
Gibberellin A12	332.19	0.045	1.21	3.69	↓^a^		0.007	1.13	1.18	↑^b^
Bis-ferulamidobutane	440.19	0.021	1.22	3.76	↓^a^		0.0017	1.25	2.14	↑^b^
Plicamine	462.17	0.015	1.25	3.89	↓^a^		0.003	1.29	2.12	↑^b^
Linoleic acid	280.24	0.0029	1.11	2.71	↓^b^		0.006	1.08	6.22	↑^b^
Deoxycholic acid	392.29	0.038	1.05	2.72	↑^a^		0.0027	1.12	2.35	↓^b^

(*n* = 3).

a *p* < 0.05.

b *p* < 0.01.

**TABLE 2:** Pathways obtained among the control, CCl_4_, and the SCU group in feces samples.

Pathway name	*p* Value	*p* Value adjusted
Metabolic pathways	0.972	0.972
Linoleic acid metabolism	0.000304	0.0131
Biosynthesis of unsaturated fatty acids	0.00219	0.0314
Bile secretion	0.000724	0.0156

**TABLE 3:** Altered metabolites in liver tissues among the control, CCl4, and the SCU group.

Metabolites	CCl_4_ vs. control group						SCU vs. CCl_4_ group			
	MW	*p* value	VIP score	Change fold	Trend		*p* Value	VIP score	Change fold	Trend
13-cis-Retinoicacid	300.44	0.020	1.29	1.69	↓^a^		0.036	1.20	2.11	↑^a^
Callystatin A	456.32	0.017	1.52	1.56	↑^a^		0.044	1.59	3.26	↓^a^
Oleamide	281.27	0.048	1.16	2.72	↑^a^		0.031	1.33	1.55	↓^a^
All-trans-retinoic acid	300.43	0.043	1.19	2.33	↓^a^		0.011	1.36	2.71	↑^a^
Retinyl ester	286.45	0.0021	1.05	3.45	↓^b^		0.0013	1.45	3.29	↑^b^
Choline	103.10	0.009	1.30	1.48	↓^b^		0.034	2.13	2.87	↑^a^
Palmitoyl ethanolamide	299.28	0.007	1.97	1.41	↓^b^		0.021	1.29	2.37	↑^a^
D-gluconic acid	196.05	0.039	2.10	2.91	↓^a^		0.0019	1.93	1.65	↑^b^
L-pyroglutamic acid	129.04	0.030	1.82	2.26	↓^a^		0.048	1.46	3.14	↑^a^
Xanthine	152.03	0.025	1.04	1.36	↓^a^		0.012	1.09	3.27	↑^a^

(*n* = 3).

a *p* < 0.05.

b *p* < 0.01.

**TABLE 4:** Pathways obtained among the control, CCl_4_, and the SCU group in liver tissues samples.

Pathway name	*p* Value	*p* Value adjusted
Metabolic pathways	0.870	0.870
Glycerophospholipid metabolism	0.0833	0.231
Neuroactive ligand-receptor interaction	0.186	0.241
Carbon metabolism	0.366	0.381
Retinol metabolism	0.00164	0.0342

## Discussion

Alcohol-, drug-, and toxin-induced liver injury can develop into liver fibrosis, cirrhosis, and even cancer, which has been deemed one of the most health-threatening diseases in the world (Hou et al., 2019; Zhu et al., 2019; Shirani et al., 2020). At present, more attention is being given to the development of anti-liver injury agents from functional foods, such as edible herbs, due to their high efficacy, multiple targets, and low side effects (Hao et al., 2020). In this study, we revealed the potential functional targets of SCU, an active flavonoid derived from the traditional Chinese herb *E. breviscapus,* through RNA-seq and gut microbiome 16 S rRNA-seq. We also confirmed that SCU exerts its robust protective effects against CCl_4_-induced liver injury. Furthermore, a nontarget metabolomics approach based on UHPLC-Q-Exactive MS/MS shows that SCU presents hepatoprotective effects on liver injury by reversing the potential biomarkers to normal levels. Collectively, SCU is a promising agent for liver injury therapy.

CCl_4_-induced mouse liver injury is a widely used animal model to mimic liver damage in humans. CCl_4_-transformed trichloromethyl radicals (CCl3•) can react with molecular oxygen to form a highly toxic trichloromethyl peroxyl radical (CCl3OO•), and these free radicals can irreversibly bind hepatic macromolecules, including DNAs, proteins, and lipids, triggering oxidative stress and promoting a cascade of damage to the liver (Amzar et al., 2017). Hepatic CYP2E1 is responsible for the biotransformation of CCl_4_, and CCl_4_-derived free radicals can increase the activity of CYP2E1, aggravating CCl_4_-induced liver injury (Yu et al., 2014). Furthermore, various substrates, such as ethanol and acetaminophen, are metabolized into hepatotoxins *via* CYP2E1 (Wang et al., 2016; Torres et al., 2019). Cho et al. (2018) underlined the pivotal role of CYP2E1 in alcohol-induced liver injury. As per the results of transcriptomic analysis, CYP2E1 is a potential functional target for SCU. To corroborate this finding, we examined the mRNA and protein expression levels of CYP2E1 by immunohistochemistry, Western blot, and RT-qPCR. Our data show that SCU significantly downregulated CYP2E1 expression in mice exposed to CCl_4_. The degree of oxidative stress in the liver was decreased when CYP2E1 was inhibited. We determined hepatic SOD activity and MDA content. SOD is the main antioxidant enzyme that eliminates these free radicals and limits hepatic damage. MDA, the final product of lipid peroxidation, is a biomarker of oxidative stress (Sobeh et al., 2020; Zhang et al., 2020). The results show that SCU significantly enhances SOD activity and attenuates MDA production relative to the CCl_4_ group.

CCl_4_-induced oxidative stress and CCl_4_ itself can lead to inflammatory responses. Inflammation is a normal immune response that repairs and returns injured tissue to a healthy state in the presence of tissue injury. Nevertheless, an excessive inflammatory response leads to overproduction of inflammatory mediators, which may, in turn, aggravate damage to the local site or even cause life-threatening disorders (Kim et al., 2020). According to the results of transcriptomic analysis, NF-κB is another potential functional target of SCU. NF-κB is a master regulator of the cellular inflammatory response. Without stimulation, NF-κB heterodimers bind to IκBα and maintain an inactive form. However, when stimulation occurs, the degradation of IκBα followed by the activation of NF-κB can contribute to the overproduction of proinflammatory cytokines, worsening hepatic damage (Zhu et al., 2015; Shin et al., 2019). To confirm this finding, we examined the IκBα and NF-κB protein expression levels in the liver and found that SCU substantially reversed CCl_4_-induced IκBα degradation and NF-κB activation. To demonstrate that the hepatic inflammatory response is decreased when the IκBα/NF-κB signaling pathway is inhibited, we detected the transcripts of IL-6, IL-1β, and TNF-α by RT-qPCR. IL-6, IL-1β, and TNF-α are the main proinflammatory mediators that can amplify inflammatory reactions, playing a crucial role in the inflammatory response, and they are regulated by the IκBα/NF-κB signaling pathway (Kwon et al., 2016). The results show that SCU intake significantly inhibits the production of proinflammatory cytokines induced by CCl_4_.

AST, ALT, and TBIL are hallmarks of liver injury. When the membranes of hepatocytes are ruptured by CCl_4_, these markers are released into the extracellular space and enter the systemic circulation, thereby increasing their serum contents (Ozer et al., 2008). Our data show that SCU significantly reduces the serum levels of AST, ALT, and TBIL, improving CCl_4_-induced liver injury. Moreover, the efficacy of a high dose of SCU is similar to that of the positive drug. The liver is the unique site of ALB synthesis, and serum ALB levels serve as a specific marker of hepatic synthetic function. However, decreased ALB levels often occur in end-stage liver diseases, such as hepatic failure, rather than short-term experimental liver injury because of its very long half-life (Ozer et al., 2008). Histopathological assays and TUNEL assays yielded consistent conclusions: SCU effectively ameliorates hepatic lesions and apoptosis. In summary, these data prove that the hepatoprotective effects of SCU are associated with inhibiting the CYP2E1 and IκBα/NF-κB signaling pathways.

Given that SCU is a natural flavonoid with very poor absorption, we infer that the beneficial effects of SCU are mainly due to modulation of the gut microbiota. Our data demonstrate a significant moderating effect of SCU on the gut microbiota. At the phylum level, a comparison of gut microbial structure between the CCl_4_ model and the SCU group revealed a trend towards a decrease in the *Bacteroidetes-to-Firmicutes* ratio, which is consistent with conclusion of Zhang et al. (2018). At the genus level, we observed that SCU treatment enriched the relative abundances of *Lactobacillus*, *Bifidobacterium*, and *Akkermansia*, which are reported to promote liver repair and improve liver injury and liver-associated diseases (Yeung et al., 2015; Deng et al., 2020; Jantararussamee et al., 2021). Moreover, *Lactobacillus* was the key genus responding to SCU treatment, according to Spearman’s correlation analysis. Mountains of evidence have proven that *Lactobacillus* supplementation or *Lactobacillus*-derived metabolites can effectively ameliorate chemical toxin-induced liver injury (Chiva et al., 2002; Saeedi et al., 2020). These data indicate that *Lactobacillus* may play a critical role in the hepatic effects of SCU. Our previous study strongly proves this conclusion. We utilized a cocktail of antibiotics to deplete and destroy the gut microbiota (especially *Lactobacillus*) in mice and found that the hepatic effects of SCU were reversed (Miao et al., 2020). *Lactobacillus* can also repress the translocation of bacteria, which has emerged as a pivotal factor in aggravating liver diseases, such as alcoholic hepatitis (Slattery et al., 2019). However, bacterial or bacterial product translocation depends on the degree of gut leakiness. In contrast to alcohol- and acetaminophen-induced liver injury, the CCl_4_-induced liver injury model had no significant impact on gut integrity (Mazagova et al., 2015). Thus, various liver injury models have to be utilized to verify these results. Mouse coculture is another way to highlight the importance of gut microbiota. We raised CCl_4_-treated mice and CCl_4_ + SCU-treated mice in the same cage, and the results demonstrated that the cocultured CCl_4_ group had a lower degree of liver injury (Supplementary Figures 4, 5). These data prove that the hepatoprotective effects of SCU are partly due to the modulation of gut microbiota.

The occurrence of liver injury is also involved in metabolic disorders. UHPLC-Q-Exactive MS/MS was utilized to analyze the fecal and liver tissue metabolic profiles of mice treated with CCl_4_ and SCU. Seven altered metabolites in feces and 11 in liver tissues were obtained, which are involved in linoleic acid metabolism, biosynthesis of unsaturated fatty acids, bile secretion, and retinol metabolism. Linoleic acid associated with linoleic acid metabolism and biosynthesis of unsaturated fatty acids was repressed in the CCl_4_-treated group. Moreover, it is well documented that CCl_4_-induced liver injury is often accompanied by steatosis, indicating that CCl_4_ can reduce the ability of gut microbiota to metabolize lipids, leading to lipid accumulation (Tsuchida et al., 2018). CCl_4_-induced lipid accumulation is significantly improved by SCU, as evidenced by the higher level of linoleic acid in a few studies. Bile secretion and retinol metabolism are tightly associated with bile acid driven by intestinal and hepatic FXR (Norum et al., 1986) and have emerged as important factors in multiple physiological and pathological states of the liver. Bile acids, such as deoxycholic acid (DCA), can inhibit intestinal FXR, thereby suppressing the transcription of FGF19/FGF15, which can reach the liver through the portal vein. FGF19/FGF15 inhibits CYP7A1 expression in the liver and then decreases bile acid synthesis (Wahlström et al., 2016). Moreover, all-trans retinoic acid involved in retinol metabolism can significantly activate hepatic FXR, contributing to the inhibition of CYP7A1, thereby decreasing the synthesis of bile acid (Zhang et al., 2020). In this study, we found an increased deoxycholic acid (DCA) level in the feces and decreased all-trans retinoic acid levels in the liver in the CCl_4_-treated group, which was improved by SCU treatment. These data indicate that SCU may maintain bile acid and lipid metabolism homeostasis to improve liver injury.

Drugging the microbiome has been deemed an attractive therapy and whether the change in the microbiome is correlative or causative to disease has become an interesting concern. In our previous and present studies, we demonstrated that the SCU-altered microbiome has a causal role in protecting against liver injury induced by CCl_4_ through antibiotic treatment and mouse coculture. Additionally, we identified robust microbiome drug targets, *Lactobacillus*. It has been proposed that probiotic *Lactobacillus* can ameliorate outcomes in a few clinically relevant models of liver injury, such as non-alcoholic fatty liver disease and alcoholic liver disease (Saeedi et al., 2020). Previous studies have shown that *Lactobacillus* is quite effective at innate inflammatory signaling pathways, including the NF-κB signaling pathway (Collier-Hyams et al., 2005). Presently, transcriptomics profiles have uncovered a prominent downregulation of NF-κB in SCU-treated mouse liver samples. We further demonstrated that *Lactobacillus* was negatively associated with NF-κB through Spearman’s correlation analysis. These results indicate that SCU may elicit its NF-κB inhibition effects partly through enriching *Lactobacillus* in a CCl_4_-induced liver injury model. A growing body of evidence suggests that the composition of gut microbiota affects systemic metabolism through alterations in the host metabolome (Nicholson et al., 2012). Manipulating the gut microbiome could reverse the dysregulation of host metabolism associated with a pathological state (Chang et al., 2015). Here, we found that a metabolic pathway involving bile acid homeostasis is strongly associated with CCl_4_-induced liver injury and actively responsive to therapeutic interventions for SCU. Indeed, Long et al. (2017) highlighted the importance of *Lactobacillus* to host health by maintaining bile acid homeostasis regulated by hepatic FXR and CYP7A1. In our study, transcriptomic profiling also revealed that SCU has a potential regulatory effect on hepatic FXR and CYP7A1. These results indicate that SCU may maintain bile acid homeostasis to protect against CCl_4_-induced liver injury partly through enriching *Lactobacillus*. This finding needs further investigation.

Considering that SCU is a flavonoid compound with poor absorption, we believe that the hepatic effects of SCU are mostly due to modulating of gut microbiota, which is pivotal in multiple phenotypes associated with liver injury. Nevertheless, it is also reported that SCU may elicit its pharmacological effects *via* isoscutellarin, the secondary metabolite transformed by gut microbiota (Wang and Ma, 2018). Gut microbiota collectively encodes 150-fold more genes than the human genome, and this genetic diversity encompasses a rich enzyme repository with drug-metabolizing potential (Zimmermann et al., 2019). Most natural compounds with low bioavailability are delivered orally and undergo chemical modifications inevitably and the resulting metabolites may have functional properties that are better than those of their parent drugs (Obach et al., 2013). However, few studies focus on this and need further research.

**FIGURE 5:**
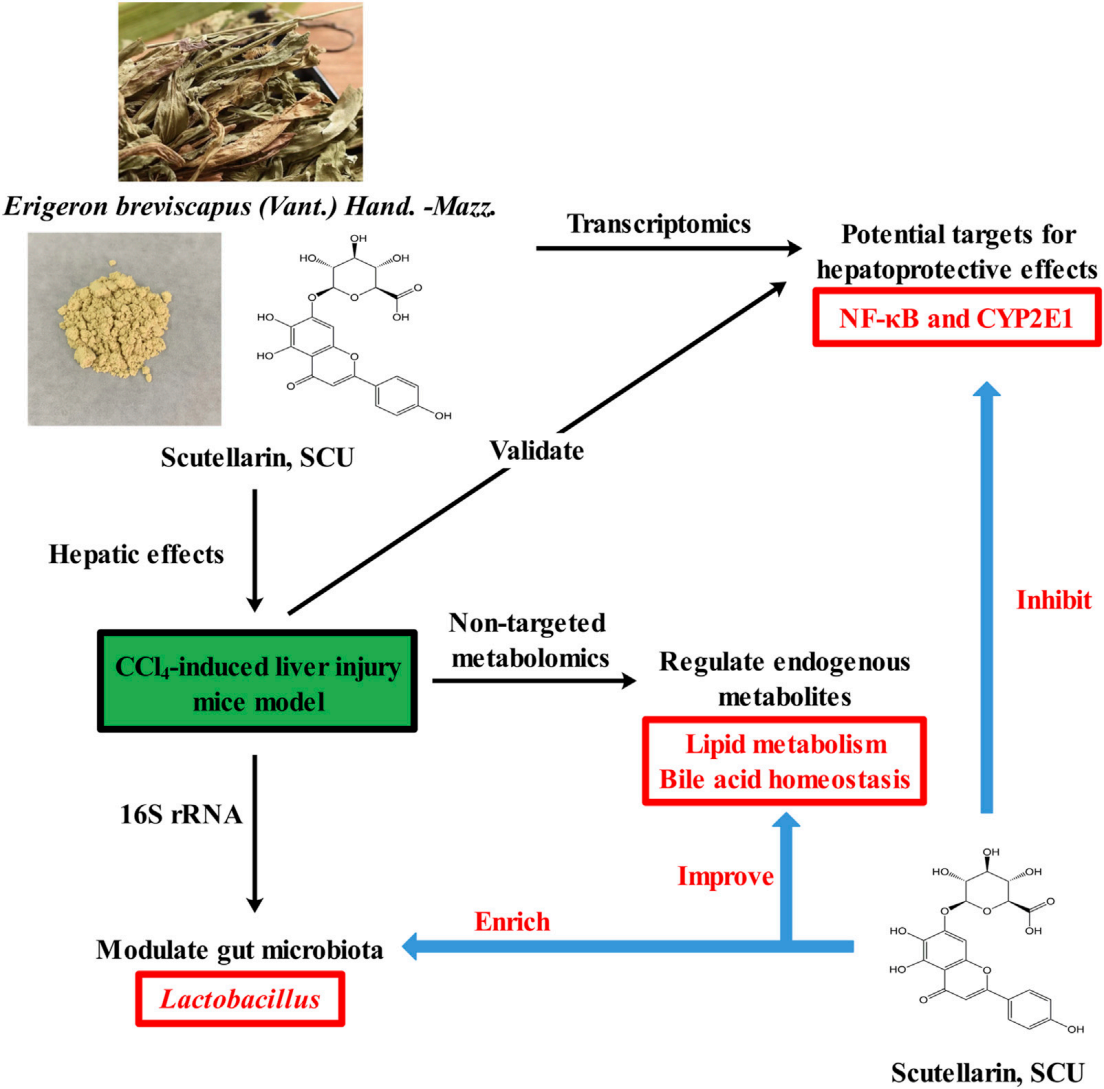
Graphical abstract of protective property of scutellarin against liver injury induced by carbon tetrachloride in mice.

In conclusion, our study demonstrated that SCU exerted robust hepatoprotective effects against CCl_4_-induced liver injury by repressing the CYP2E1 and IκBα/NF-κB signaling pathways, modulating the gut microbiota, enriching *Lactobacillus*, and regulating endogenous metabolites involved in lipid metabolism and bile acid homeostasis. Our study suggests that SCU is a potential candidate for the development of functional food for the treatment of liver injury (Figure 5).

## Data Availability

The datasets presented in this study can be found in online repositories. The names of the repository/repositories and accession number(s) can be found in the article/Supplementary Material.
