# Supplementary material for: Protective Property of Scutellarin Against Liver Injury Induced by Carbon Tetrachloride in Mice
Source: Front Pharmacol. 2021 Aug 5;12:710692. doi: 10.3389/fphar.2021.710692 (PMC8374867; doi:10.3389/fphar.2021.710692)
Supplement: Supplementary file 1 [file DataSheet1.DOCX]

**Protective property of scutellarin against liver injury induced by carbon tetrachloride in mice**

Zhimin Miao^#^, Yong Lai^*#^, Yingying Zhao^#^, Lingmin Chen, Jianeng Zhou, Chunyan Li and Yan Wang^*^

College of Pharmacy, Dali University, Dali 671000, China.

^#^These authors contributed equally to this work

^*^Corresponding author

Yong Lai, E-mail laiyong8879@163.com

Yan Wang, E-mail jessica9428@sina.com


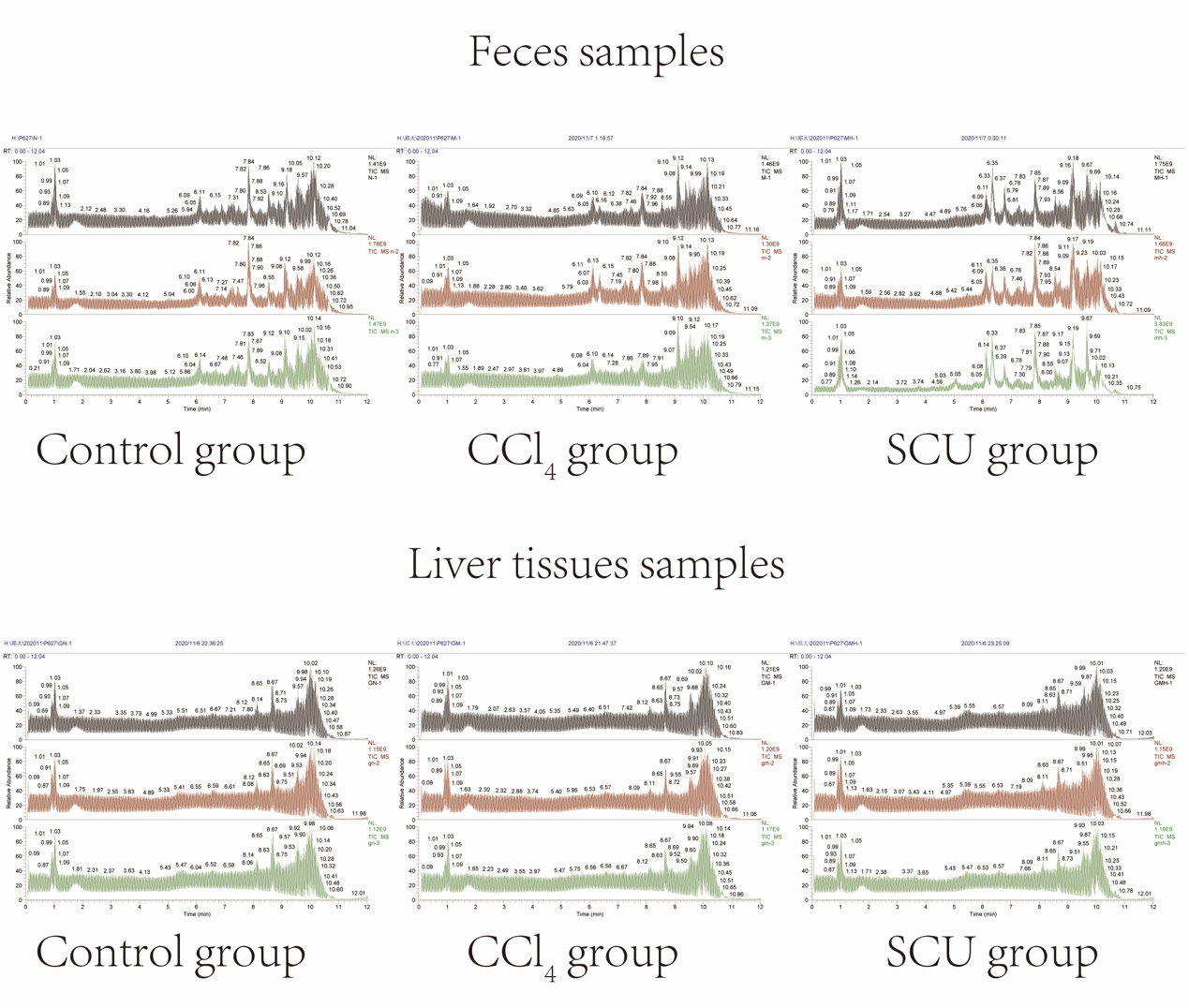


Figure S1 the total ion current (TIC) of feces and liver tissues samples in all groups.


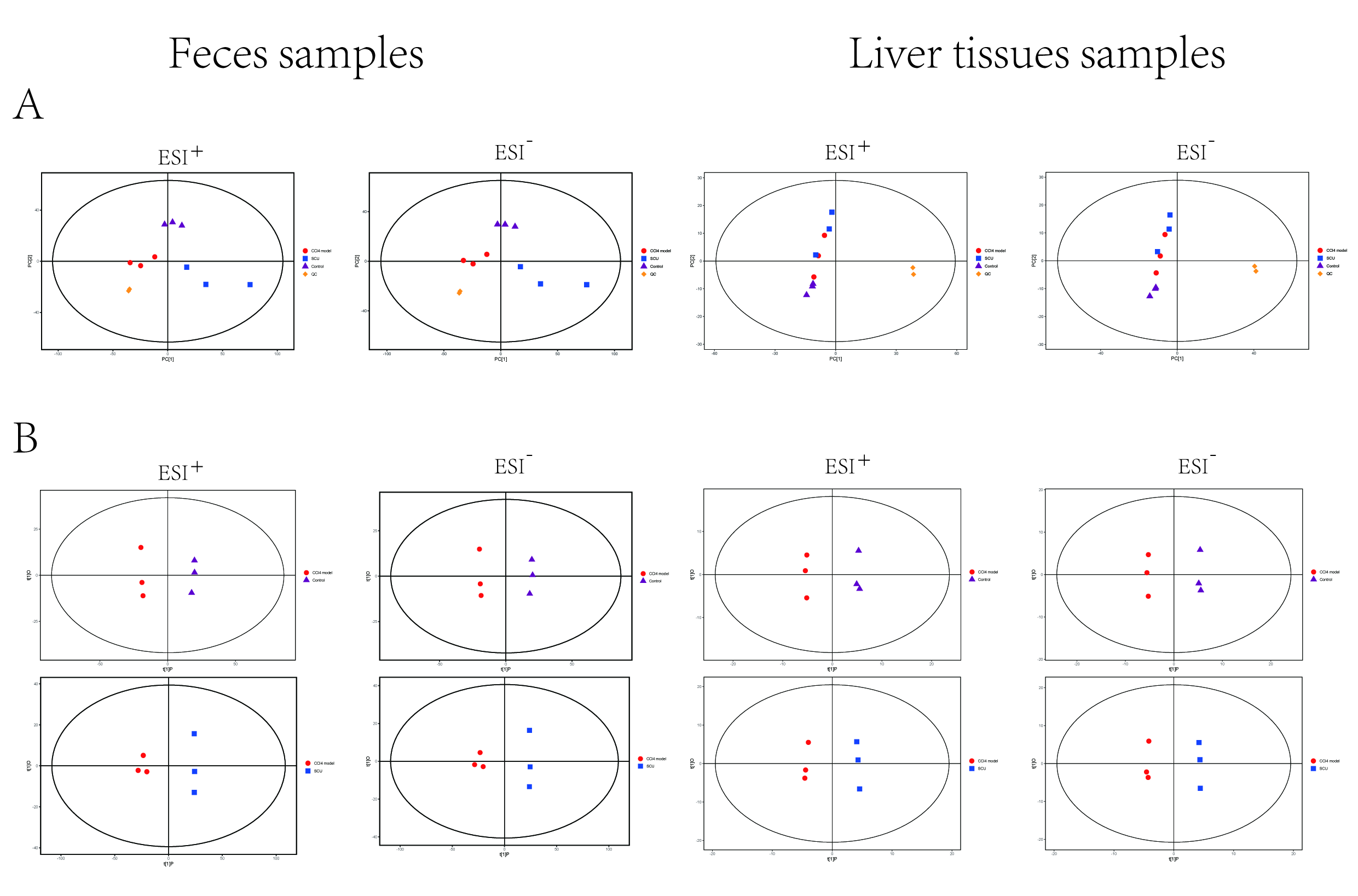


Figure S2 the PCA (A) and OPLS-DA (B) of feces and liver tissues samples in all groups. PCA: R^2^X=0.840 (ESI+)/0.838 (ESI-) in feces samples. PCA: R^2^X=0.800 (ESI+)/0.807 (ESI-) in liver tissues samples. OPLS-DA (feces samples): R^2^X=0.751, R^2^Y=0.998, Q^2^=0.934 (Control-CCl4 model); R^2^X=0.853, R^2^Y=0.990, Q^2^=0.846 (CCl4 model-SCU, ESI+); R^2^X=0.748, R^2^Y=0.999, Q^2^=0.953 (Control-CCl4 model); R^2^X=0.849, R^2^Y=0.990, Q^2^=0.851 (CCl4 model-SCU, ESI-); OPLS-DA (liver tissues samples): R^2^X=0.637, R^2^Y=0.999, Q^2^=0.914 (Control-CCl4 model); R^2^X=0.613, R^2^Y=0.997, Q^2^=0.767 (CCl4 model-SCU, ESI+); R^2^X=0.631, R^2^Y=0.999, Q^2^=0.914 (Control-CCl4 model); R^2^X=0.590, R^2^Y=0.999, Q^2^=0.764 (CCl4 model-SCU, ESI-);


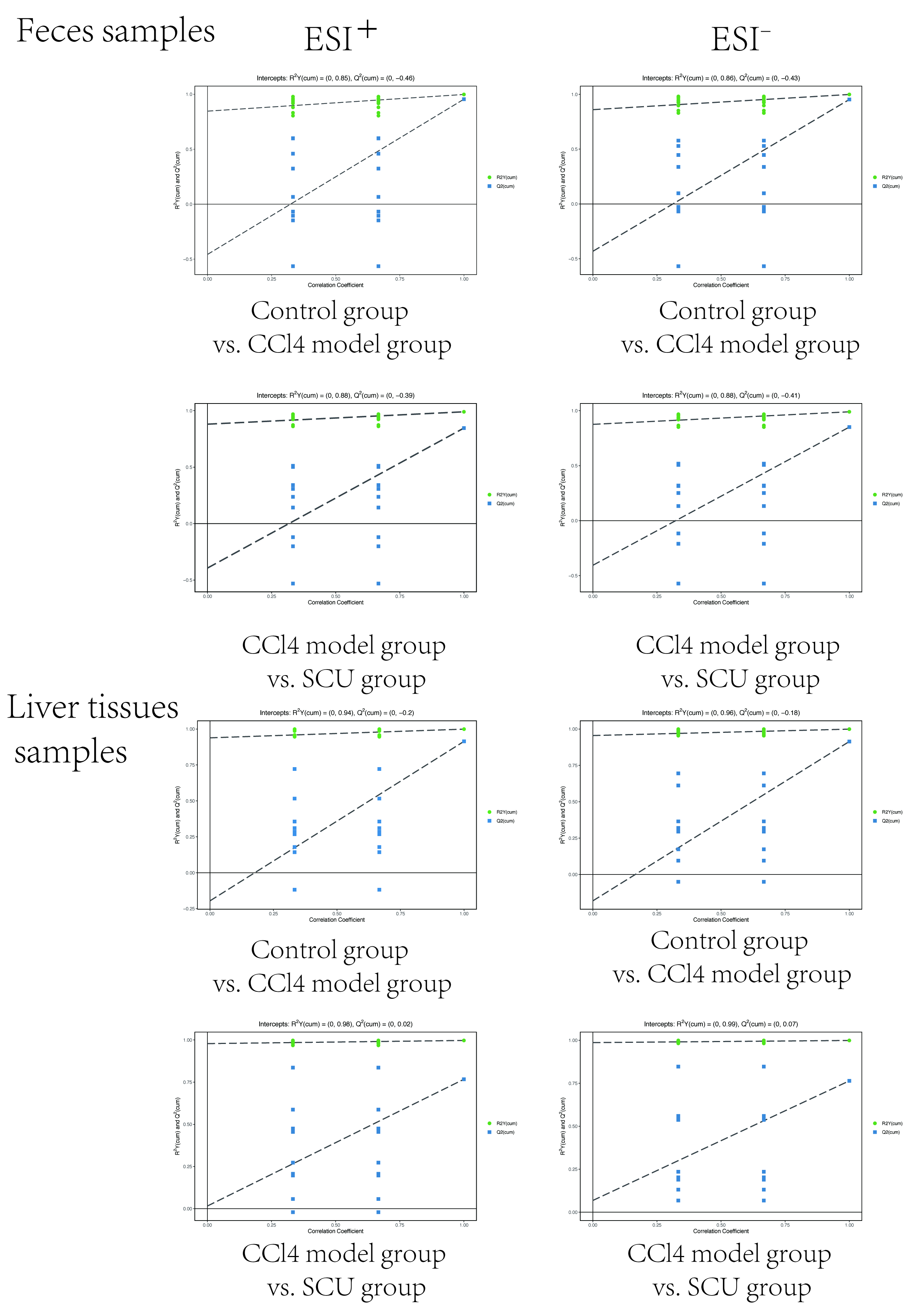


Figure S3 permutation tests in feces samples and liver tissues samples


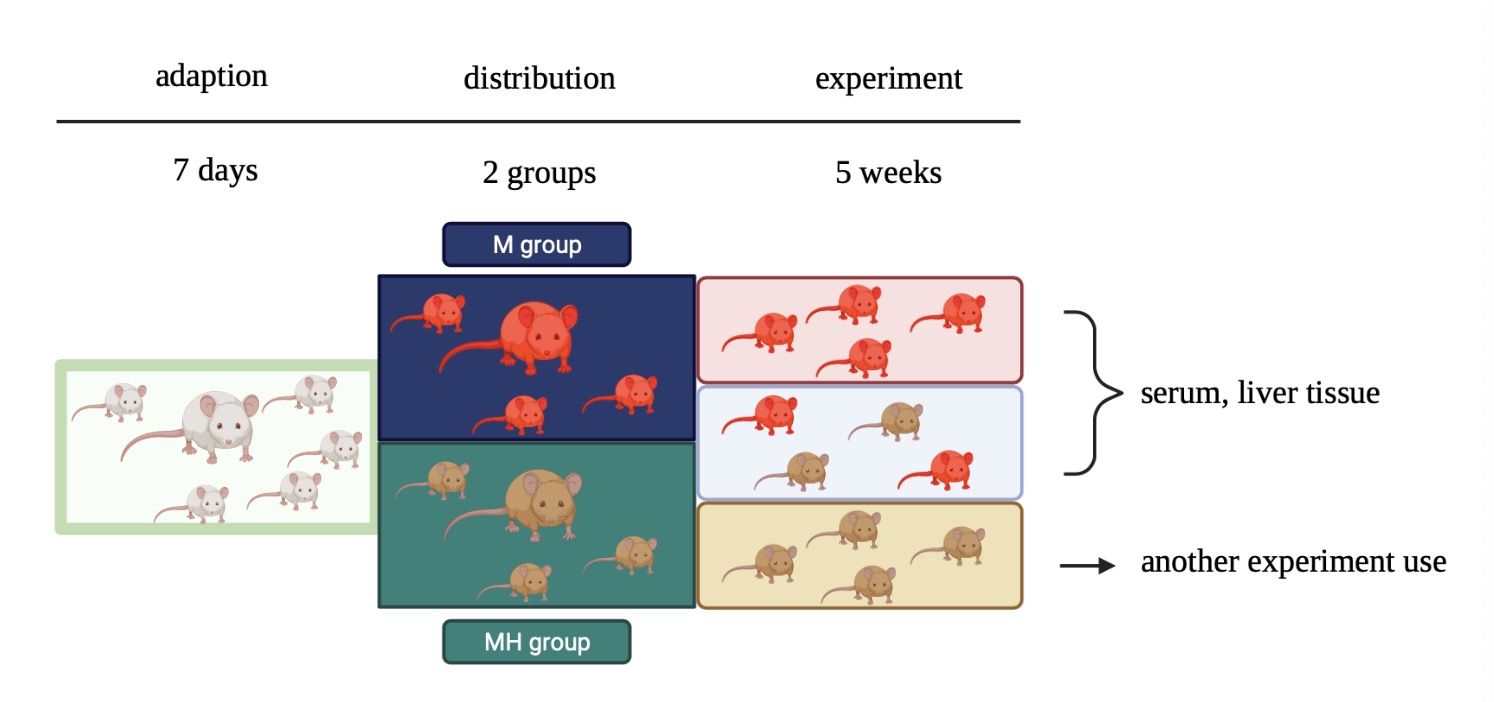


Figure S4 Experimental design of co-culture. M: CCl_4_-treated group. MH: CCl_4_ + 0.12 mmol/kg SCU-treated group.


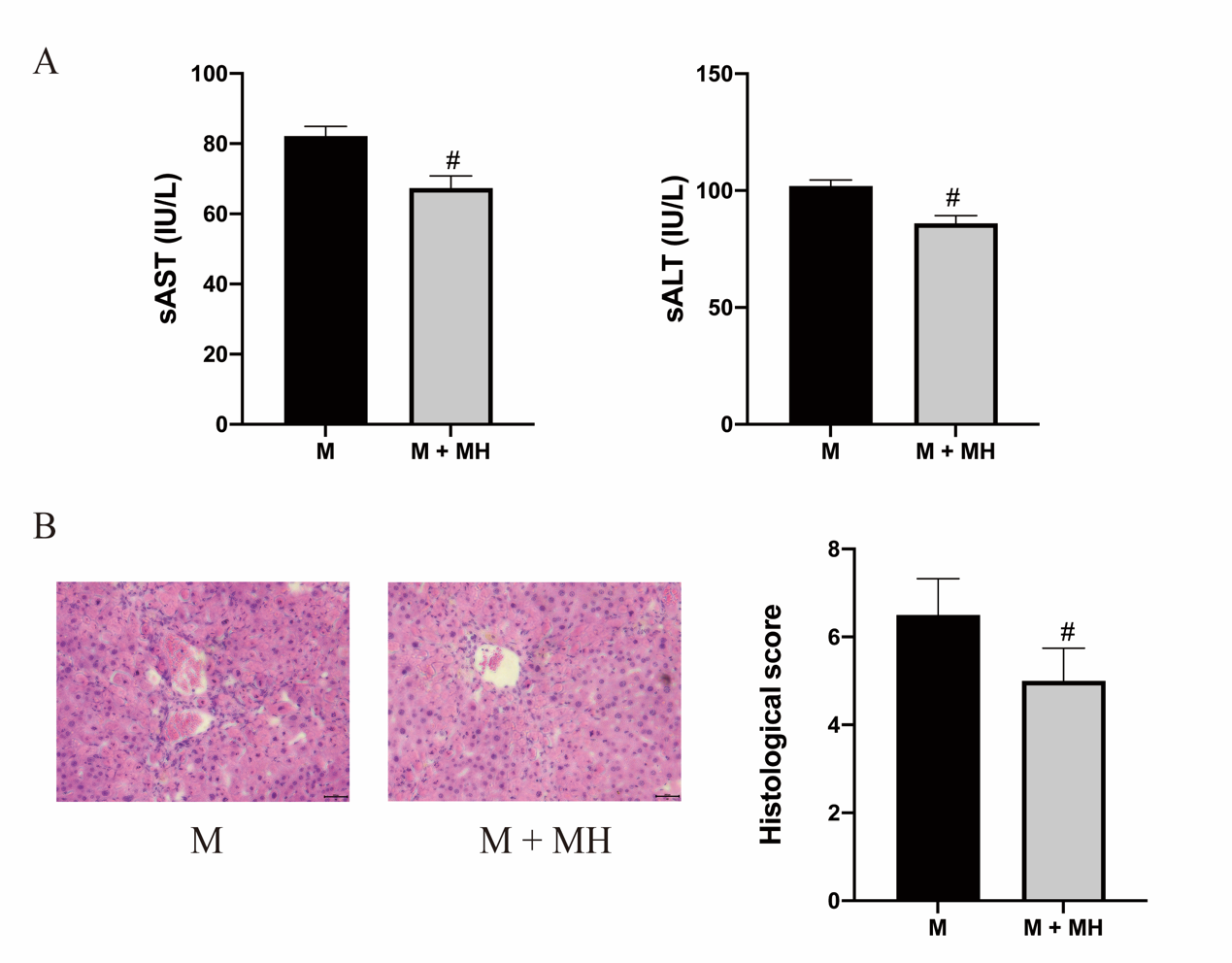


Figure S5 The results of co-culture experiment.
